# Genetic Variants of IκB Kinase β (*IKBKB*) and Polymerase β (*POLB*) Were Not Associated with Systemic Lupus Erythematosus Risk in a Chinese Han Population

**DOI:** 10.1371/journal.pone.0132556

**Published:** 2015-07-13

**Authors:** Yuan Li, Ziyan Wu, Shulan Zhang, Si Chen, Ping Li, Jing Li, Chongwei Cao, Bin Liu, Fengchun Zhang, Yongzhe Li

**Affiliations:** 1 Department of Rheumatology, the Affiliated Hospital of Qingdao University, Qingdao, China; 2 Department of Rheumatology and Clinical Immunology, Peking Union Medical College Hospital, Chinese Academy of Medical Sciences and Peking Union Medical College, Key Laboratory of Rheumatology and Clinical Immunology, Ministry of Education, Beijing, China; University of Birmingham, UNITED KINGDOM

## Abstract

A previous large-scale replication study validation of a genome wide association study (GWAS) identified IκB kinase β (*IKBKB*) single nucleotide polymorphisms (SNPs) as a risk factor associated with systemic lupus erythematosus (SLE) in a Chinese Han population. *IKBKB* SNPs were associated with polymerase β (*POLB)* SNPs and reduced POLB expression, and this was proposed to be an underlying cause of human SLE development. In the current case-control study, we evaluated *IKBKB* (rs12676482 and rs2272733) and *POLB* (rs3136717 and rs3136744) SNPs in 946 SLE patients and 961 healthy controls. We investigated the possible association of these four SNPs with SLE in a Chinese Han population using the polymerase chain reaction-ligation detection reaction (PCR-LDR) technique. The differences in the frequencies of the four SNP alleles and the genotypes and haplotypes of the *POLB* polymorphisms were statistically insignificant when the SLE patients were compared with the controls in the Chinese Han population enrolled in this study (all, *p* ˃ 0.05). Furthermore, no associations were detected using different genetic models (additive, dominant, and recessive; all, *p* ˃ 0.05). Our findings indicate that the *IKBKB* (rs12676482 and rs2272733) and *POLB* (rs3136717, rs3136744) SNPs confer no genetic predisposition to SLE risk in this Chinese Han population.

## Introduction

Systemic lupus erythematosus (SLE) is a chronic systemic autoimmune disease that causes the immune system to attack healthy tissues and organs through the production of a diverse array of autoantibodies, complement activation and immune complex formation [1]. SLE prevalence ranges from 0.031% to 0.07% in China and from 0.007% to 0.071% in Europe [2,3]. SLE affects more women than men (a ratio of 9:1, female to male), especially during the childbearing years [4]. Although the etiology and pathogenesis of SLE remain unknown, it is possible that environmental and epigenetic factors contribute to SLE [5]. SLE frequently develops in people with a family history of the disease, and a number of likely candidate genes have been investigated. For instance, the HLA class I, II and III genes have been associated with SLE development [6]. Additionally, the X chromosome carries immunological related genes, and, thus, it is possible that X chromosome mutations could contribute to SLE onset. However, no single causal gene has yet been conclusively identified. Thus, further investigation of SLE genetic susceptibility could provide a better understanding of SLE pathogenesis and help identify novel strategies for effective control of this chronic autoimmune disease.

IκB kinase β (*IKBKB*) encodes IKK-β protein, a cytokine-activated intracellular immune response signaling component that acts *via* activation of the canonical NF-κB signaling pathway. The rs12676482 SNP was recently localized in *IKBKB*, and identified and significantly associated with SLE in a large-scale replication study based on a genome wide association study (GWAS) of a Chinese Han population [7]. Notably, rs12676482 is in perfect linkage disequilibrium with rs2272733 (r^2^ = 1), which is highly associated with decreased *POLB* expression [8].

Environmental factors may induce DNA damage, and this damage is normally recognized and repaired by complex mechanisms in cells [9,10]. However, recent studies have reported that DNA repair pathway genes may not work properly in SLE patients [11,12]. Therefore, DNA damage may induce the immune system to attack and induce the apoptosis of the affected cells and tissues. Indeed, SNPs in DNA repair genes have been associated with a predisposition to develop the autoantibodies and clinical manifestations of SLE [13,14].

*POLB*, which was localized to chromosome 8p11.2, encodes a DNA polymerase (Pol-β) that is involved in the repair of single-strand breaks as a part of the base excision and repair (BER) pathway [15]. In one recent study, a *POLB* ^Y265C/C^ mouse model developed an autoimmune pathology that strongly resembled SLE [16]. The *POLB* ^Y265C/C^ mice exhibited symptoms that included dermatitis, antinuclear antibodies (ANAs) and glomerular nephritis. The study proposed that decreased Pol-β activity during the generation of immune diversity led to lupus-like disease in mice, and suggested that decreased Pol-β expression in humans may be an underlying cause of SLE [16]. After examining the HapMap Phase II CHB data (Hapmap Data Rel 27 PhaseII + III, Feb 111 09, on NCBI 36 assembly, dbSNP126) (http://hapmap.ncbi.nlm.nih.gov/), we located two tag SNPs in the POLB region. Other DNA repair gene SNPs may predispose individuals to the development of particular clinical and laboratory features. One study reported that serine/threonine kinase 17A (STK17A), another DNA repair-related gene, was associated with SLE susceptibility [17]. TREX1 polymorphisms have been negatively associated with autoantibodies in SLE [18]. The XRCC1 Arg399Gln polymorphism was significantly associated with the presence of anti-dsDNA antibody [19].

Because SLE patients may have deficient DNA repair, and because *POLB* is involved in DNA damage repair, we hypothesized that the *POLB* gene may be a candidate SLE susceptibility gene and analyzed its association with SLE. Therefore, we conducted a case-control study examining the potential associations of *IKBKB* (rs12676482 and rs2272733) and *POLB* (rs3136717 and rs3136744) with SLE risk in a Chinese Han population.

## Methods

### Study population

We recruited 946 SLE patients and 961 healthy controls from the Rheumatology Department of Beijing Union Medical College Hospital (Beijing, China). All patients met the 1997 American College of Rheumatology (ACR) classification criteria for SLE [20]. All patients and controls were unrelated individuals of Chinese Han ethnicity. This study was approved by the Ethics Committee of the Beijing Union Medical College Hospital. All participants signed a written informed consent form.

### SNP selection, DNA extraction and genotyping

The four SNPs selected for this study were selected based on findings reported by previous studies and a lupus-prone mouse study [16]. Specifically, the *IKBKB* (rs12676482 and rs2272733) and *POLB* (rs3136717 and rs3136744) SNPs were selected according to the HapMap Phase II CHB database (Hapmap Data Rel 27 Phase II + III; Feb 11, 09; NCBI 36 assembly; dbSNP126; http://hapmap.ncbi.nlm.nih.gov/) by Haploview v4.2. Criteria were minor allele frequency (MAF) > 0.05 and LD values r^2^ > 0.8.

Genomic DNA was extracted from 2 mL ethylenediaminetetraacetic acid (EDTA) anticoagulated peripheral blood samples using DNA isolation kits (Bioteke, Beijing, China) according to the manufacturer’s instructions and stored at -80°C until use. SNP genotyping was conducted using the polymerase chain reaction-ligation detection reaction (PCR-LDR) method [21,22] with technical support from Biowing Applied Biotechnology Company (Shanghai, China). Target DNA sequences were amplified using a multiplex PCR method, and the probe and primer sequences used for *POLB* and *IKBKB* detection are shown in S1 and S2 Tables. The ligation reaction for each SNP was carried out in a 20 μl reaction mixture containing 2 μl PCR buffer, 0.6 μl Mg^2+^, 2 μl dNTP, 0.2 μl Qiagen HotStarTaq Polymerase (QIAGEN, Hilden, Germany), 4 μl Q-solution, 2 μl of each primer and 12.2 μl H_2_O. DNA amplification was performed using a Perkin-Elmer Gene Amp PCR Systems 9600 thermocycler (Waltham, MA, USA) and the following protocol: initial denaturation at 95°C for 15 min, 35 cycles of denaturation at 94°C for 30 s, annealing at 56°C for 90 s, extension at 72°C for 60 s and final extension at 72°C for 7 min. The LDR was performed in a 10 μl reaction mixture containing 1 μl buffer, 1 μl probe mix, 0.05 μl Taq DNA ligase (New England Biolabs, Ipswich, MA, USA), 4 μl multi-PCR product and 4 μl H_2_O. The LDR reaction protocol was: 35 cycles of denaturation at 95°C for 2 min, annealing at 94°C for 30 s and extension at 60°C for 2 min. The LDR fluorescent products were sequenced using an ABI 377 sequencer (Forster city, CA, USA). For quality control, genotyping was repeated for 50 blinded blood samples.

### Statistical analysis

Each SNP was assessed in the patient and control populations for departure from Hardy-Weinberg equilibrium (HWE) using the Chi-square (χ^2^) test. SNPs that deviated from HWE (*p* < 0.05) were excluded from further analysis. The age distribution between the case and control groups was assessed with the Mann-Whitney U test. The χ^2^ test was used to compare the allele and genotype frequencies between the case and control groups using PLINKv1.07 (http://pngu.mgh.harvard.edu/Bpurcell/plink/). Haplotype analysis was carried out using Haploview software v4.2 (http://www.broadinstitute.org/haploview). Odds ratio (OR) was calculated with exact confidence intervals (CI) of 95%, and a *p* value < 0.05 was considered statistically significant. Three logistic regression models (additive, dominant and recessive) were used to analyze SNPs.

## Results

### Characterization of study population

SLE patients presented with a variety of different blood autoantibodies, including anti-nuclear antibodies (ANA), anti-SSA/B, anti-Sm, anti-RNP and anti-dsDNA. These autoantibodies were assessed using indirect immunofluorescence or double immunodiffusion methods. The SLE patients had a mean age of 36.26 ± 12.97 years and consisted of 84 males and 862 females. The healthy control individuals had a mean age of 36.78 ± 10.90 years and consisted of 87 males and 874 females (Table 1). The Mann-Whitney U test showed that the ages were well matched between the case and control groups (*p* > 0.05). Patients and controls were enrolled from the same geographic location.

**Table 1:** Characteristics of SLE patients and health control subjects.

Characteristics	Case (%)	Control (%)
Male/female	84/862	87/874
Age, years (mean ± s.d.)	36.26 ± 12.97	36.78 ± 10.90
ANA	928 (98.1)	-
anti-SSA antibody	461 (48.7)	-
anti-SSB antibody	107 (11.3)	-
anti-Sm antibody	186 (19.7)	-
anti-RNP antibody	276 (29.2)	-
anti-dsDNA antibody	449 (47.4)	-
Low C3 or C4 level	601 (63.5)	-
Nephritis	518 (54.8)	-
Neuropsychiatric disorder	140 (14.8)	-

### Allele and genotype frequencies between cases and controls

The four SNPs selected for this study were evaluated with a call rate more than 99% in all 946 SLE patients and 961 healthy controls. No deviation from Hardy-Weinberg equilibrium was observed in the controls (*p* > 0.05). The allelic and genotypic frequency of the four SNPs is presented in Table 2. The data indicate no significant differences between the case and control groups (all, *p* > 0.05), indicating no significant association between the tested polymorphisms and SLE predisposition. Statistical analysis using multiple logistic regressions in three genetic models also showed no significant difference between the SLE patients and healthy controls (Table 3).

**Table 2:** Allele and genotype distribution of these four SNPs in SLE patients and healthy controls.

Gene	SNP	Allele frequency				Genotype frequency			
		Allele	Case/control (n)	P value	OR (95% CI)	Genotype	Case/control (n)	P value	χ^2^
IKBKB	rs12676482	A	198/198	0.60	1.06 (0.84–1.33)	A/A	12/8	0.59	216.9
		G	1648/1748			A/G	174/182		
						G/G	737/783		
IKBKB	rs2272733	T	204/212	0.93	1.01 (0.81–1.26)	T/T	12/10	0.84	94.07
		C	1642/1734			T/C	180/192		
						C/C	731/771		
POLB	rs3136717	C	178/177	0.83	1.02 (0.82–1.28)	C/C	12/10	0.89	0.22
		T	1668/1699			C/T	154/157		
						T/T	757/771		
POLB	rs3136744	C	152/140	0.38	1.11 (0.88–1.41)	C/C	9/6	0.61	0.98
		A	1694/1736			C/A	134/128		
						A/A	780/804		

**Table 3:** Analysis of these four SNPs based on three genetic models.

Gene	SNP	Additive model		Dominant model		Recessive model	
		P	OR (95%CI)	P	OR (95%CI)	P	OR (95%CI)
IKBKB	rs12676482	0.61	1.06 (0.85–1.33)	0.78	1.02 (0.80–1.29)	0.31	1.27 (0.55–2.96)
IKBKB	rs2272733	0.93	1.01 (0.81–1.26)	0.94	0.99 (0.78–1.26)	0.58	1.27 (0.55–2.96)
POLB	rs3136717	0.83	1.02 (0.83–1.27)	0.92	1.01 (0.80–1.28)	0.64	1.22 (0.53–2.84)
POLB	rs3136744	0.39	1.11 (0.88–1.41)	0.44	1.10 (0.85–1.42)	0.42	1.53 (0.54–4.31)

### Association between SNPs and SLE phenotypes

Next, we investigated the potential associations of these SNPs with the clinicopathological data of the SLE patients. First, we stratified the patients into eight SLE sub-phenotype groups, including SLE patients who were positive for anti-SSA/B, anti-Sm, anti-RNP, or anti-dsDNA antibodies, or who had low complement levels, nephritis or neurological disorders. We compared positive patients with negative patients, positive patients with all healthy controls and negative patients with all healthy controls. However, we found no associations between the examined SNPs and any of the clinicopathological data from the SLE patients (Table 4).

**Table 4:** Association of these four SNPs genotype frequencies with various clinical features.

Subphenotypes	Comparison	rs12676482 (IKBKB)		rs2272733 (IKBKB)		rs3136717 (POLB)		rs3136744 (POLB)	
		P	OR (95% CI)	P	OR (95% CI)	P	OR (95% CI)	P	OR (95% CI)
Anti- SSA	P (n = 461) vs. N (n = 485)	0.15	1.08 (0.89–1.31)	0.28	0.84 (0.61–1.15)	0.23	0.83 (0.61–1.13)	0.10	0.75 (0.54–1.06)
	P (n = 461) vs. C (n = 961)	0.66	0.94 (0.70–1.25)	0.56	0.92 (0.70–1.21)	0.59	0.93 (0.70–1.22)	0.77	0.96 (0.70–1.30)
	N (n = 485) vs. C (n = 961)	0.21	1.19 (0.91–1.56)	0.49	1.10 (0.84–1.43)	0.39	1.12 (0.86–1.45)	0.10	1.27 (0.96–1.68)
Anti-SSB	P (n = 107) vs. N (n = 839)	0.08	1.49 (0.95–2.33)	0.12	1.42 (0.91–2.22)	0.06	1.51 (0.98–2.32)	0.08	1.50 (0.96–2.38)
	P (n = 107) vs. C (n = 961)	0.07	1.50 (0.97–2.33)	0.16	1.37 (0.88–2.13)	0.08	1.46 (0.95–2.24)	0.05	1.58 (1.00–2.50)
	N (n = 839) vs. C (n = 961)	0.96	0.99 (0.78–1.24)	0.75	0.96 (0.76–1.21)	0.80	0.97 (0.77–1.22)	0.68	1.05 (0.82–1.35)
Anti- Sm	P (n = 186) vs. N (n = 760)	0.94	1.01 (0.69–1.49)	0.85	0.96 (0.66–1.41)	0.77	0.94 (0.64–1.40)	0.95	0.99 (0.65–1.50)
	P (n = 186) vs. C (n = 961)	0.75	1.07 (0.72–1.58)	0.90	0.97 (0.66–1.44)	0.91	0.98 (0.66–1.44)	0.64	1.10 (0.73–1.66)
	N (n = 760) vs. C (n = 961)	0.63	1.06 (0.83–1.35)	0.88	1.02 (0.81–1.29)	0.76	1.04 (0.82–1.31)	0.40	1.12 (0.87–1.44)
Anti-RNP	P (n = 276) vs. N (n = 670)	0.55	1.11 (0.78–1.52)	0.78	1.05 (0.74–1.48)	0.76	1.05 (0.75–1.47)	0.80	1.05 (0.73–1.51)
	P (n = 276) vs. C (n = 961)	0.43	1.14 (0.82–1.59)	0.79	1.04 (0.75–1.44)	0.71	1.06 (0.77–1.46)	0.43	1.15 (0.81–1.63)
	N (n = 670) vs. C (n = 961)	0.82	1.03 (0.80–1.32)	0.97	0.99 (0.78–1.27)	0.94	1.01 (0.79–1.28)	0.50	1.10 (0.84–1.43)
Anti-dsDNA	P (n = 449) vs. N (n = 497)	0.24	0.82 (0.60–1.14)	0.26	0.83 (0.60–1.15)	0.30	0.84 (0.62–1.16)	0.16	0.78 (0.56–1.10)
	P (n = 449) vs. C (n = 961)	0.76	0.96 (0.71–1.28)	0.54	0.91 (0.69–1.21)	0.64	0.94 (0.71–1.24)	0.87	0.97 (0.72–1.33)
	N (n = 497) vs. C (n = 961)	0.28	1.16 (0.89–1.51)	0.49	1.10 (0.84–1.43)	0.45	1.11 (0.85–1.43)	0.13	1.24 (0.94–1.64)
Complement	P (n = 601) vs. N (n = 345)	0.24	1.23 (0.87–1.74)	0.23	1.23 (0.88–1.73)	0.51	1.11 (0.81–1.54)	0.66	1.08 (0.76–1.53)
	P (n = 601) vs. C (n = 961)	0.31	1.14 (0.89–1.47)	0.53	1.08 (0.85–1.39)	0.62	1.07 (0.83–1.36)	0.32	1.14 (0.87–1.50)
	N (n = 345) vs. C (n = 961)	0.64	0.93 (0.67–1.28)	0.44	0.88 (0.64–1.21)	0.77	0.96 (0.70–1.30)	0.74	1.06 (0.76–1.47)
Nephritis	P (n = 518) vs. N (n = 431)	0.18	1.25 (0.90–1.74)	0.11	1.30 (0.94–1.80)	0.05	1.37 (0.99–1.88)	0.11	1.32 (0.94–1.86)
	P (n = 517) vs. C (n = 961)	0.25	1.17 (0.90–1.52)	0.36	1.13 (0.87–1.46)	0.23	1.17 (0.91–1.50)	0.11	1.25 (0.95–1.65)
	N (n = 431) vs. C (n = 961)	0.65	0.93 (0.69–1.26)	0.34	0.87 (0.64–1.17)	0.29	0.85 (0.64–1.14)	0.73	0.95 (0.69–1.30)
Neuropsychiatric disorder	P (n = 140) vs. N (n = 806)	0.23	0.74 (0.45–1.21)	0.24	0.75 (0.46–1.22)	0.33	0.79 (0.50–1.26)	0.54	0.86 (0.53–1.40)
	P (n = 140 vs. C (n = 961)	0.42	0.83 (0.52–1.33)	0.33	0.79 (0.49–1.27)	0.45	0.84 (0.53–1.33)	0.92	0.97 (0.60–1.59)
	N (n = 806) vs. C (n = 961)	0.40	1.11 (0.87–1.40)	0.68	1.05 (0.83–1.32)	0.63	1.06 (0.84–1.33)	0.31	1.14 (0.89–1.46)

Abbreviation: P, patients positive for a certain phenotype; N, patients negative for a certain phenotype; C, controls

### Haplotype analysis of SNPs and SLE

We also performed linkage disequilibrium (LD) analysis to assess whether any *POLB* or *IKBKB* haplotype was associated with SLE risk. Three major haplotypes (T-A, C-C and C-A) were resolved from these two SNPs, and their frequencies showed no statistically significant differences when SLE patients were compared with controls (Table 5).

**Table 5:** Haplotype analysis of POLB SNPs.

Gene	SNPs	Haplotype	Total frequency	Case (%)	Control (%)	χ^2^	P value
POLB	rs3136717-rs3136744	TA	0.905	90.4	90.6	0.038	0.846
	rs3136717-rs3136744	CC	0.078	8.2	7.4	0.736	0.391
	rs3136717-rs3136744	CA	0.017	1.5	2.0	1.742	0.187

## Discussion

A previous large-scale replication study showed that *IKBKB* rs12676482 was the susceptibility locus for SLE development in the Chinese Han population [7]. rs12676842 is a SNP in the noncoding region adjacent to *POLB* that exists in perfect linkage disequilibrium with rs2272733 (r^2^ = 1), which is highly associated with reduced *POLB* expression in human cells [8]. Unfortunately, our current study failed to confirm association of these SNPs with SLE development. The reason for this discrepancy is unclear; however, it might be due to the relatively low power of the sample size for detecting the small effect described in the original report at a significance level of 0.05. Therefore, we re-calculated the sample size based on the MAF of these SNPs (ca. 0.1) and found that 29,579 cases would be needed to obtain sufficient statistical power, suggesting that the current sample size is too small to conclusively associate these SNPs with SLE development. Various ethnic and environmental factors are known to influence SLE susceptibility. The SLE patients in the previous cohort came from central and southern China, and our SLE patients come from central and northern China. Therefore, further study with a larger sample size is needed to verify the association, or lack of association, between the SNPs examined in this study and SLE risk.

SLE has a variety of clinical and pathological manifestations. This variety may be an indication that ethnic background plays a substantial role in SLE risk. Genetics and epigenetics are both involved in determining the phenotypes of developing organisms, and further study is needed to better understand the effect of genes with allelic or genetic heterogeneity on disease risk [23]. It is possible that the clinical heterogeneity of SLE and the differences observed between the study population and other Han Chinese samples involve these parallel systems. Epigenetics is the study of transmissible and reversible changes in gene expression that are not accompanied with alterations in nucleotide sequences. Epigenetic information is carried chiefly by DNA itself, histones and noncoding RNAs. Examining the epigenetics of different SLE populations may shed light on the extraordinary complexity of gene regulation and expression involved in the heterogeneity of SLE. Different genetic backgrounds from different ancestries and various populations may result in different genetic risk factors for SLE. Recent studies have reported that DNA repair gene mutations are associated with immunological processes, and that these mutations could lead to the development of autoimmune disease [24–26]. DNA repair protein deficiencies have been investigated in SLE patients [27–30]. Additionally, the *POLB* ^Y265C/C^ mouse model developed an autoimmune pathology that strongly resembled human SLE, including dermatitis, antinuclear antibodies and glomerular nephritis. It is known that POLB protein maintains genome integrity *via* DNA base excision repair. Furthermore, POLB deficiency results in cellular hypersensitivity to alkylating agent treatment, which manifests as the induction of apoptosis and chromosomal breaking [31]. Our current investigation of the *POLB* SNPs showed no association between rs3136717 or rs3136744 and SLE risk. Although the results were negative, the current study is, to the best of our knowledge, the first to attempt to associate *POLB* SNPs with SLE risk.

During development and disease progression of SLE, various autoantibodies are present in the sera of most SLE patients, including ANA, anti-dsDNA, anti-SSA/B, anti-Sm, and anti-RNP antibodies. These antibodies could serve as diagnostic markers or disease severity indicators and could be used to clinically assess the diverse SLE phenotypes. Our current cohort of SLE patients were diagnosed and assessed using the 1997 American College of Rheumatology (ACR) SLE diagnosis criteria [20]. Among these criteria, autoantibodies (such as anti-Smith, anti-dsDNA, anti-phospholipid or ANA antibodies) are considered to have high sensitivity and specificity [20]. The body’s ability to make autoantibodies to chromatin, nucleosomes and DNA may be influenced by individual genetics [18]. Insufficient DNA damage repair may contribute to the immune dysfunction of SLE patients; however, the BER pathway may have a different role in the pathogenesis of autoimmune diseases in the mouse model than in human autoimmune disease. Another possible explanation the results we observed in this study is that rs12676482 may regulate genes other than *POLB*. *POLB* is downstream of *IKBKB*; however, other susceptibility genes and risk factors may affect SLE *via* the regulation of POLB expression. It is unknown how the *POLB* rs3136744 SNP predisposes SLE patients to generate particular autoantibodies; therefore, additional studies that are more detailed will be required to determine which molecular mechanisms are controlled by *POLB* genetic variants.

This study has some inherent limitations that should be considered when interpreting our findings. First, we only assessed two tagged SNPs each for *IKBKB* and *POLB*. The results obtained by this study may not completely represent the association between these SNPs and SLE risk and, therefore, the examination of more loci is needed to verify the association of the *IKBKB* and *POLB* SNPs with SLE. Second, because of the significant ethnic differences and population heterogeneity of SLE patients worldwide, it remains important to determine whether *IKBKB* SNPs or *POLB* SNPs are associated with SLE in multiple populations. In conclusion, this study indicates that *IKBKB* (rs12676482 and rs2272733) and *POLB* (rs3136717 and rs3136744) SNPs confer no genetic predisposition for SLE in a Han Chinese population.

## Supporting Information

S1 Table Primers of target genes used in the PCR. (DOCX)

S2 Table LDR probe sequences. (DOC)

## References

[1] Mok CC, Lau CS. Pathogenesis of systemic lupus erythematosus. J Clin Pathol. [Journal Article; Review]. 2003 2003-7-01;56(7):481–90. 12835292 10.1136/jcp.56.7.481 PMC1769989

[2] Zeng QY, Chen R, Darmawan J, Xiao ZY, Chen SB, Wigley R, et al Rheumatic diseases in China. Arthritis Res Ther. [Journal Article; Research Support, Non-U.S. Gov't]. 2008 2008-1-20;10(1):R17 10.1186/ar2368 18237382 PMC2374446

[3] Danchenko N, Satia JA, Anthony MS. Epidemiology of systemic lupus erythematosus: a comparison of worldwide disease burden. Lupus. [Comparative Study; Journal Article; Review]. 2006 2006-1-20;15(5):308–18. 16761508 10.1191/0961203306lu2305xx

[4] Rahman A, Isenberg DA. Systemic lupus erythematosus. N Engl J Med. [Journal Article; Review]. 2008 2008-2-28;358(9):929–39. 10.1056/NEJMra071297 18305268

[5] Moser KL, Kelly JA, Lessard CJ, Harley JB. Recent insights into the genetic basis of systemic lupus erythematosus. Genes Immun. [Journal Article; Research Support, N.I.H., Extramural; Research Support, Non-U.S. Gov't; Research Support, U.S. Gov't, Non-P.H.S.; Review]. 2009 2009-7-01;10(5):373–9. 10.1038/gene.2009.39 19440199 PMC3144759

[6] Martens HA, Nolte IM, van der Steege G, Schipper M, Kallenberg CG, Te MG, et al An extensive screen of the HLA region reveals an independent association of HLA class I and class II with susceptibility for systemic lupus erythematosus. Scand J Rheumatol. [Comparative Study; Journal Article]. 2009 2009-1-20;38(4):256–62. 10.1080/03009740802552469 19255932

[7] Sheng YJ, Gao JP, Li J, Han JW, Xu Q, Hu WL, et al Follow-up study identifies two novel susceptibility loci PRKCB and 8p11.21 for systemic lupus erythematosus. Rheumatology (Oxford). [Journal Article; Research Support, Non-U.S. Gov't]. 2011 2011-4-01;50(4):682–8. 21134959 10.1093/rheumatology/keq313

[8] Zeller T, Wild P, Szymczak S, Rotival M, Schillert A, Castagne R, et al. Genetics and beyond—the transcriptome of human monocytes and disease susceptibility. PLoS One. [Journal Article; Research Support, Non-U.S. Gov't]. 2010 2010-01-20;5(5):e10693. 8. Zhou BB, Elledge SJ. The DNA damage response: putting checkpoints in perspective. Nature. [Journal Article; Review]. 2000 2000-11-23;408(6811):433–9. 10.1371/journal.pone.0010693 PMC2872668 20502693

[9] Zhou BB, Elledge SJ. The DNA damage response: putting checkpoints in perspective. Nature. [Journal Article; Review]. 2000 2000-11-23;408(6811):433–9 11100718 10.1038/35044005

[10] Friedberg EC. DNA damage and repair. Nature. [Comment; Journal Article; Review]. 2003 2003-1-23;421(6921):436–40. 12540918 10.1038/nature01408

[11] Warchol T, Mostowska A, Lianeri M, Lacki JK, Jagodzinski PP. XRCC1 Arg399Gln gene polymorphism and the risk of systemic lupus erythematosus in the Polish population. Dna Cell Biol. [Journal Article; Research Support, Non-U.S. Gov't]. 2012 2012-1-01;31(1):50–6. 10.1089/dna.2011.1246 21682595 PMC3246417

[12] Bassi C, Xavier D, Palomino G, Nicolucci P, Soares C, Sakamoto-Hojo E, et al Efficiency of the DNA repair and polymorphisms of the XRCC1, XRCC3 and XRCC4 DNA repair genes in systemic lupus erythematosus. Lupus. [Journal Article; Research Support, Non-U.S. Gov't]. 2008 2008-11-01;17(11):988–95. 10.1177/0961203308093461 18852222

[13] Hur JW, Sung YK, Shin HD, Park BL, Cheong HS, Bae SC. TREX1 polymorphisms associated with autoantibodies in patients with systemic lupus erythematosus. Rheumatol Int. [Journal Article; Research Support, Non-U.S. Gov't]. 2008 2008-6-01;28(8):783–9. 18092167 10.1007/s00296-007-0509-0

[14] Lin YJ, Lan YC, Wan L, Huang CM, Lin CW, Hsueh KC, et al The NBS1 genetic polymorphisms and the risk of the systemic lupus erythematosus in Taiwanese patients. J Clin Immunol. [Journal Article; Research Support, Non-U.S. Gov't]. 2010 2010-9-01;30(5):643–8. 10.1007/s10875-010-9427-0 20571895

[15] Dogliotti E, Fortini P, Pascucci B, Parlanti E. The mechanism of switching among multiple BER pathways. Prog Nucleic Acid Res Mol Biol. [Journal Article; Review]. 2001 2001-1-20;68:3–27. 11554307 10.1016/s0079-6603(01)68086-3

[16] Senejani AG, Liu Y, Kidane D, Maher SE, Zeiss CJ, Park HJ, et al Mutation of POLB causes lupus in mice. Cell Rep. [Journal Article; Research Support, N.I.H., Extramural]. 2014 2014-1-16;6(1):1–8. 10.1016/j.celrep.2013.12.017 24388753 PMC3916967

[17] Da SFA, de Azevedo SJ, Pancotto JA, Donadi EA, Segat L, Crovella S, et al Polymorphisms in STK17A gene are associated with systemic lupus erythematosus and its clinical manifestations. Gene. [Journal Article; Research Support, Non-U.S. Gov't; Review]. 2013 2013-9-25;527(2):435–9. 10.1016/j.gene.2013.06.074 23860322

[18] Hur JW, Sung YK, Shin HD, Park BL, Cheong HS, Bae SC. TREX1 polymorphisms associated with autoantibodies in patients with systemic lupus erythematosus. Rheumatol Int. [Journal Article; Research Support, Non-U.S. Gov't]. 2008 2008-6-01;28(8):783–9. 18092167 10.1007/s00296-007-0509-0

[19] Bassi C, Xavier D, Palomino G, Nicolucci P, Soares C, Sakamoto-Hojo E, et al Efficiency of the DNA repair and polymorphisms of the XRCC1, XRCC3 and XRCC4 DNA repair genes in systemic lupus erythematosus. Lupus. [Journal Article; Research Support, Non-U.S. Gov't]. 2008 2008-11-01;17(11):988–95. 10.1177/0961203308093461 18852222

[20] Hochberg MC. Updating the American College of Rheumatology revised criteria for the classification of systemic lupus erythematosus. Arthritis Rheum. [Letter]. 1997 1997-9-01;40(9):1725. 10.1002/art.1780400928 9324032

[21] Thomas G, Sinville R, Sutton S, Farquar H, Hammer RP, Soper SA, et al Capillary and microelectrophoretic separations of ligase detection reaction products produced from low-abundant point mutations in genomic DNA. Electrophoresis. [Journal Article; Research Support, Non-U.S. Gov't; Research Support, U.S. Gov't, P.H.S.]. 2004 2004-6-01;25(10–11):1668–77. 15188256 10.1002/elps.200405886

[22] Yi P, Chen Z, Zhao Y, Guo J, Fu H, Zhou Y, et al PCR/LDR/capillary electrophoresis for detection of single-nucleotide differences between fetal and maternal DNA in maternal plasma. Prenat Diagn. [Journal Article; Research Support, Non-U.S. Gov't]. 2009 2009-3-01;29(3):217–22 10.1002/pd.2072 19177453

[23] Miceli-Richard C. Epigenetics and lupus. Joint Bone Spine. [Journal Article]. 2015 2015-3-01;82(2):90–3. 10.1016/j.jbspin.2014.03.004 25523441

[24] Warchol T, Mostowska A, Lianeri M, Lacki JK, Jagodzinski PP. XRCC1 Arg399Gln gene polymorphism and the risk of systemic lupus erythematosus in the Polish population. Dna Cell Biol. [Journal Article; Research Support, Non-U.S. Gov't]. 2012 2012-1-01;31(1):50–6. 10.1089/dna.2011.1246 21682595 PMC3246417

[25] Koyama A, Kubota Y, Shimamura T, Horiuchi S. Possible association of the X-ray cross complementing gene 1 (XRCC1) Arg280His polymorphism as a risk for rheumatoid arthritis. Rheumatol Int. [Comparative Study; Journal Article; Research Support, Non-U.S. Gov't]. 2006 2006-6-01;26(8):749–51. 16284769 10.1007/s00296-005-0066-3

[26] Graessler J, Verlohren M, Graessler A, Zeissig A, Kuhlisch E, Kopprasch S, et al Association of chondromodulin-II Val58Ile polymorphism with radiographic joint destruction in rheumatoid arthritis. J Rheumatol. [Comparative Study; Journal Article]. 2005 2005-9-01;32(9):1654–61. 16142856

[27] Courtney PA, Crockard AD, Williamson K, Irvine AE, Kennedy RJ, Bell AL. Increased apoptotic peripheral blood neutrophils in systemic lupus erythematosus: relations with disease activity, antibodies to double stranded DNA, and neutropenia. Ann Rheum Dis. [Journal Article; Research Support, Non-U.S. Gov't]. 1999 1999-5-01;58(5):309–14. 10225817 10.1136/ard.58.5.309 PMC1752888

[28] McCurdy D, Tai LQ, Frias S, Wang Z. Delayed repair of DNA damage by ionizing radiation in cells from patients with juvenile systemic lupus erythematosus and rheumatoid arthritis. Radiat Res. [Journal Article; Research Support, Non-U.S. Gov't]. 1997 1997-1-01;147(1):48–54. 8989369

[29] McConnell JR, Crockard AD, Cairns AP, Bell AL. Neutrophils from systemic lupus erythematosus patients demonstrate increased nuclear DNA damage. Clin Exp Rheumatol. [Journal Article]. 2002 2002-9-01;20(5):653–60. 12412196

[30] Mandel M, Gurevich M, Pauzner R, Kaminski N, Achiron A. Autoimmunity gene expression portrait: specific signature that intersects or differentiates between multiple sclerosis and systemic lupus erythematosus. Clin Exp Immunol. [Journal Article]. 2004 2004-10-01;138(1):164–70. 15373920 10.1111/j.1365-2249.2004.02587.x PMC1809188

[31] Narayan S, He F, Wilson SH. Activation of the human DNA polymerase beta promoter by a DNA-alkylating agent through induced phosphorylation of cAMP response element-binding protein-1. J Biol Chem. [Journal Article; Research Support, U.S. Gov't, P.H.S.]. 1996 1996-8-02;271(31):18508–13 8702497 10.1074/jbc.271.31.18508

