# Correction: Genetic Variants of IκB Kinase β (*IKBKB*) and Polymerase β (*POLB*) Were Not Associated with Systemic Lupus Erythematosus Risk in a Chinese Han Population

**DOI:** 10.1371/journal.pone.0135606

**Published:** 2015-08-11

**Authors:** Yuan Li, Ziyan Wu, Shulan Zhang, Si Chen, Ping Li, Jing Li, Chongwei Cao, Bin Liu, Fengchun Zhang, Yongzhe Li

The first and second author affiliations are switched. The affiliations should appear as shown here:

Yuan Li^1, 2^, Ziyan Wu^1^, Shulan Zhang^1^, Si Chen^1^, Ping Li^1^, Jing Li^1^, Chunwei Cao^1^, Bin Liu^2^, Fengchun Zhang^1^, Yongzhe Li^1^

1 Department of Rheumatology and Clinical Immunology, Peking Union Medical College Hospital, Chinese Academy of Medical Sciences & Peking Union Medical College, Key Laboratory of Rheumatology and Clinical Immunology, Ministry of Education, Beijing, China,

2 Department of Rheumatology, The Affiliated Hospital of Qingdao University, China
