# Supplementary material for: Genetic Variants of IκB Kinase β (IKBKB) and Polymerase β (POLB) Were Not Associated with Systemic Lupus Erythematosus Risk in a Chinese Han Population
Source: PLoS One. 2015 Jul 13;10(7):e0132556. doi: 10.1371/journal.pone.0132556 (PMC4500405; doi:10.1371/journal.pone.0132556)
Supplement: S1 Table — (DOCX) [file pone.0132556.s001.docx]

**Supporting Information**

**S1 Table. Primers of target genes used in the PCR**

| Primer name | sequence(5’-3’) | | PCR length |
| --- | --- | --- | --- |
| rs12676482 | AAGCAAGGGCCTAGCTTGAG | AGCCACAGACAGGATTATG | 82 |
| rs2272733 | AACCAACCATCTTTGTACCC | CATTCCATAGAGAACAGGTG | 82 |
| rs3136717 | TTTTGGTCTGGCCCTTGGAG | CGAGAACTGTTTTCTGGAAG | 100 |
| rs3136744 | TTCACAAGTAACCTAAGGAG | GGATAACTTAGAGATGAGAC | 90 |
