# Supplementary material for: Genetic Variants of IκB Kinase β (IKBKB) and Polymerase β (POLB) Were Not Associated with Systemic Lupus Erythematosus Risk in a Chinese Han Population
Source: PLoS One. 2015 Jul 13;10(7):e0132556. doi: 10.1371/journal.pone.0132556 (PMC4500405; doi:10.1371/journal.pone.0132556)
Supplement: S2 Table — (DOC) [file pone.0132556.s002.doc]

**Supporting Information**

S2 Table. LDR probe sequences

| Probe | Sequence (5’-3’) | LDR product |
| --- | --- | --- |
| rs12676482_modify | P-GCATATTTGCATCTTTCATAATCCTTTTTTTTTTTTTTTTTTTTTTTTTTTTTTTTTTTTTTTTTTTTTTTTTTTTTTT-FAM |  |
| rs12676482_A | TTTTTTTTTTTTTTTTTTTTTTTTTTTTTTTTTTTTTTTTTTTTTTTTTTTTTTTTTTTTTTTTTTTTTTTTTTTTTTTTTGGAGGGAGGTCATGTGCCATGTTTT | 185 |
| rs12676482_G | TTTTTTTTTTTTTTTTTTTTTTTTTTTTTTTTTTTTTTTTTTTTTTTTTTTTTTTTTTTTTTTTTTTTTTTTTTTTTTTTTTTGGAGGGAGGTCATGTGCCATGTTTC | 187 |
| rs2272733_modify | P-AGGCTGAGATAGAGGTTAGAAGGGTTTTTTTTTTTTTTTTTTTTTTTTTTTTTTTTTTTTTTTTTTTTTTTTTTTTTTT-FAM |  |
| rs2272733_C | TTTTTTTTTTTTTTTTTTTTTTTTTTTTTTTTTTTTTTTTTTTTTTTTTTTTTTTTTTTTTTTTTTTTTTTTTTTTTTTTTTTTTCAGGTGGAAAGAGAAATGGTCCAAG | 190 |
| rs2272733_T | TTTTTTTTTTTTTTTTTTTTTTTTTTTTTTTTTTTTTTTTTTTTTTTTTTTTTTTTTTTTTTTTTTTTTTTTTTTTTTTTTTTTTTTCAGGTGGAAAGAGAAATGGTCCAAA | 192 |
| rs3136717_modify | P-AGACAGTGACCACCCGTTTCCTCCATTTTTTTTTTTTTTTTTTTTTTTTTTTTTTTTTTTTTTTTTTTTTTTTTTTTTT-FAM |  |
| rs3136717_C | TTTTTTTTTTTTTTTTTTTTTTTTTTTTTTTTTTTTTTTTTTTTTTTTTTTTTTTTTTTTTTTTTTGGAAGGGGATATGGCAGCCTCTAAG | 170 |
| rs3136717_T | TTTTTTTTTTTTTTTTTTTTTTTTTTTTTTTTTTTTTTTTTTTTTTTTTTTTTTTTTTTTTTTTTTTTGGAAGGGGATATGGCAGCCTCTAAA | 172 |
| rs3136744_modify | P-GAAGATGTCTCATCTCTAAGTTATCTTTTTTTTTTTTTTTTTTTTTTTTTTTTTTTTTTTTTTTTTTTTTTTTTTTTTT-FAM |  |
| rs3136744_A | TTTTTTTTTTTTTTTTTTTTTTTTTTTTTTTTTTTTTTTTTTTTTTTTTTTTTTTTTTTTTTTTTTTTTTAGTAATAGGTGAATAACAGAGTAACT | 175 |
| rs3136744_C | TTTTTTTTTTTTTTTTTTTTTTTTTTTTTTTTTTTTTTTTTTTTTTTTTTTTTTTTTTTTTTTTTTTTTTTTAGTAATAGGTGAATAACAGAGTAACG | 177 |
